# Knowledge, attitudes, practices, and barriers reported by patients receiving diabetes and hypertension primary health care in Barbados: a focus group study

**DOI:** 10.1186/1471-2296-12-135

**Published:** 2011-12-02

**Authors:** O Peter Adams, Anne O Carter

**Affiliations:** 1 Faculty of Medical Sciences, The University of the West Indies, Cave Hill Campus, St. Michael, Barbados; 2 Department of Community Health and Epidemiology, Queen's University, Kingston, Ontario, Canada

## Abstract

**Background:**

Deficiencies in the quality of diabetes and hypertension primary care and outcomes have been documented in Barbados. This study aimed to explore the knowledge, attitudes and practices, and the barriers faced by people with diabetes and hypertension in Barbados that might contribute to these deficiencies.

**Methods:**

Five structured focus groups were conducted for randomly selected people with diabetes and hypertension.

**Results:**

Twenty-one patients (5 diabetic, 5 hypertensive, and 11 with both diseases) with a mean age of 59 years attended 5 focus group sessions.

Patient factors that affected care included the difficulty in maintaining behaviour change. Practitioner factors included not considering the "whole person" and patient expectations, and not showing enough respect for patients. Health care system factors revolved around the amount of time spent accessing care because of long waiting times in public sector clinics and pharmacies. Society related barriers included the high cost and limited availability of appropriate food, the availability of exercise facilities, stigma of disease and difficulty taking time off work.

Attendees were not familiar with guidelines for diabetes and hypertension management, but welcomed a patient version detailing a place to record results, the frequency of tests, and blood pressure and blood glucose targets. Appropriate education from practitioners during consultations, while waiting in clinic, through support and education groups, and for the general public through the schools, mass media and billboards were recommended.

**Conclusions:**

Primary care providers should take a more patient centred approach to the care of those with diabetes and hypertension. The care system should provide better service by reducing waiting times. Patient self-management could be encouraged by a patient version of care guidelines and greater educational efforts.

## Background

It has been estimated that in people ≥ 40 years of age in Barbados the prevalence of diabetes mellitus and hypertension is at least 19% and 55% respectively [1,2]. For people ≥ 18 years of age, 56% of men and 64% of women are either overweight or obese [3], with many such persons underestimating their body mass, being happy with their body image, and desiring little weight loss [4].

Primary care of diabetes and hypertension is delivered through public sector polyclinics at no cost, or by private general practitioners for a fee (Table 1). Patients in both sectors are provided, at no cost under the Barbados Drug Benefit Service, with an appropriate range of medications to treat diabetes and hypertension. A maximum one-month supply of medication can be dispensed at one time.

**Table 1:** Health Care setting in Barbados

Public sector	Private Sector
Eight publicly funded polyclinics strategically located around the island	At least 89 private general practitioners in 2005. Mainly solo or small group practice

Free comprehensive primary care	Service provided for a fee

Several nurses at each clinic.	Usually no nurse

A dietician and podiatrist are available on specific days	Usually no dietician or podiatrist within the clinic

Medication for diabetes and hypertension provided at no cost to the patient	Medication for diabetes and hypertension provided at no cost to the patient in 2005

Guidelines have been produced to help ensure a uniform and acceptable quality of care [5-9]. Audits of diabetes and hypertension care in Barbados have, however, indicated that deficiencies exist in the quality of care and achievement of outcome targets [10,11]. The reasons for this have not been explored.

Patient, practitioner, health care system and society related factors can all contribute to the less than ideal adoption of guidelines [12]. Biomedical approaches are not the only determinants of health care outcomes, especially with chronic disease where self-management and sustained lifestyle changes are important. Person-centred approaches to build trust and to take into account patients' ideas, concerns and expectations are important in influencing behaviour and achieving outcomes. Continuous, integrated and comprehensive care are important aspects of primary care [13].

The objectives of this study were to explore the reasons for the less than optimal outcomes observed for those with diabetes and hypertension in Barbados by holding focus groups of residents with these diseases to discuss their knowledge, attitudes, practices and the barriers they face in dealing with their diseases.

## Methods

### Focus group recruiting

Adults ≥ 40 years of age were randomly selected from the January 2004 Barbados voters' register. Telephone numbers were obtained from the telephone directory (almost all Barbadian households have a landline telephone [14]), and selected people were contacted to determine if they had diabetes mellitus or hypertension. Those who responded positively were, after an explanation of the focus group process and goals of the study, invited to attend a focus group. Random selection of voters continued until 10-12 persons with diabetes and/or hypertension agreed to participate in each focus group. It was hoped that at least 8 persons would actually attend each focus group. Five focus groups of patients were held, with 2 focusing on diabetes, 2 on hypertension and 1 on both diseases. All patient focus groups were held at Queen Elizabeth Hospital, a central point in Barbados easily accessible by public transit as well as car.

### Focus group process

A guide was prepared to conform to standard structured focus group methodology (Table 2) [15]. It was pilot tested on a convenience sample of patients. Only a few minor changes were needed. Once finalised, the guide was followed closely, but flexibility was allowed if new concepts or problems arose during a focus group session.

**Table 2:** Patient focus group guide

Domain	Topic
Knowledge	⇒ Of their disease (diabetes and/or hypertension) ⇒ Guidelines and protocols ⇒ Sources of knowledge

Attitudes	⇒ Towards their disease ⇒ Towards the care received ⇒ Of their families and communities

Practices	⇒ Managing and coping with their disease

Barriers	⇒ Faced when caring for themselves ⇒ In day-to-day life because of their disease ⇒ Recommendations for changes within and outside the health care sector that would help them achieve better health.

Each focus group session was attended by 2 investigators. One (AOC) acted as the facilitator and one (OPA) as the recorder. Participants were presented with a written sheet describing the focus group process, the goals and objectives of the study, and explaining that sessions would be taped but participants would remain anonymous. Participants' questions were answered, they were asked to sign a consent form, and then to complete an anonymous short questionnaire concerning their demographic details. The facilitator started the session by reiterating the goals and the focus group process and explaining again the use of the tape recorder. Participants were again given an opportunity to ask questions. The facilitator then started the session questions, following the guide. The recorder took notes of the discussion. When the moderator's questions were completed, participants were thanked for their contribution and asked if they had any additional comments that they wished to make. When all of the participants indicated that they had no further comments, the session ended. Following each focus group session, a debriefing session was held to summarize findings, identify any problems and develop plans for future sessions. No revisions or additions needed to be made to the guide after the sessions had started.

### Data analysis

Transcriptions of the tapes were made, and then the text was divided into sections dealing with each topic of interest. If there was difficulty understanding the tapes, the notes taken at the session were consulted. Each comment was given a content code to designate the content issue contained in the comment.

### Ethical approval

Approval was obtained from the Institutional Review Board of the University of the West Indies, Cave Hill Campus and the Ministry of Health, Barbados.

## Results

Attending the patient sessions were 5 patients with diabetes only (1 male, 4 female, mean age 52), 5 patients with hypertension only (1 male, 4 females, mean age 55) and 11 patients with both diseases (2 male, 9 female, mean age 63). Focus group size ranged from 2 to 8 persons. The mean duration of diagnosed disease for the group was 12 years, the overall mean age was 59 years and 81% were female. The onset of diabetes was ≥ 30 years of age for all attendees, and over 95% of people with diabetes in the Caribbean have type 2 [8]. It was therefore assumed that all attendees with diabetes had type 2.

### Knowledge, attitudes and practices

Persons had various concerns about their condition. One patient questioned why she could not be taken off her diabetes medication as her blood sugar was controlled for a while. Another person appreciated that hypertension was usually asymptomatic. ".... Hypertension is the silent killer because I know my pressure was high and I felt well and good."

Patients did not always adhere to medication because of side effects that they had experienced. Some patients who had experienced a hypoglycaemic episode were particularly concerned afterwards. "I tell she I am not taking that 100 'cause I took it already and it made my sugar so low, it made me sick. I said that I can take 50 but I am not going to take 100.....100 is going to send my blood sugar right down and I don't know when I am (going) to collapse." One person felt that he had to eat a bit more than the dietician recommended as he was on insulin. "You eat a little bit more to help neutralise the insulin."

### Quality of Care

Some but not all patients had nothing but praise for the quality of care they received at public sector polyclinics.

1. The *attitude of staff *was praised. "They are very helpful especially the nurses, the doctors, it is encouraging when you go to them." "I get pretty good treatment up there (when) I see the doctor and we have a rapport and they try to help, you know."

2. *Patient expectations for a physical examination were met*. ".... (at) my 3-month check up they tested my heart, toes, feet. I have no complaints I get excellent treatment."

3. *Adequate information was shared with patients*. "My doctor he has a laptop and he cannot give me no medication unless he shows me what is going on..... He shows me my medication; he shows me the side effects of my medication."

### Barriers to maintaining health

Barriers identified by attendees fell into the categories of factors - patient, practitioner, health care system and society.

#### Barriers identified by patients as being related to themselves

*Behaviour control could be difficult.* Dietary habits formed over a lifetime were difficult to change. "All your life you've been eating things that are sweet, you use sugar, you use all these different things before you become diabetic, it just don't go away." Stimulus control was a problem for some. "Sometimes you know you are not supposed to eat something but you just see it so you want it and you go and eat it, and then after you feel bad". Some practised moderation. "Sometimes I definitely does go off but I don't eat a lot. I does take a taste you know why, it does send me stark raving mad if I don't take that taste". Maintaining an exercise programme, and consistent medication taking could also be difficult.

#### Barriers identified by patients as being related to practitioners

1. The *attitude of staff *at public sector polyclinics. "There are some nurses that when you go to them, they feel that they are God, they feel on top of the world you shouldn't come and ask them nothing and when you come to them they think you are begging."

2. *Practitioners concentrating on the disease and not understanding the whole person*. "They will tell you well um your sugar is a bit high I will give you another tablet. They did not ask you what time you eat this morning...listen what happen? Did you have a problem today? What is happening? ... they don't know what type of life you live, they don't know what kind of...I am saying ok you are busy people, but at least at some time find out what you are dealing with. Find out what type of person that you are dealing with, not just hand them tablets."

3. *Problems with continuity of care *when patients see a different doctor. "My doctor change and this other doctor come on. She did not take time to read back my history right to find out what was really wrong with me, but she just order 2 more extra tablets for me." Lack of continuity at times resulted in conflicting advice and patient confusion. Some of these problems arose as many patients switch between public and private providers of their own accord disrupting continuity of care.

4. *Practitioners do not meet patient expectations for a physical examination and laboratory investigation*. "They don't really examine you at all. I haven't had an examination now in 2-3 years at the clinic. They weigh me, test the urine and get the medication, that's all." But some patients don't understand the benefits of some aspects of self-management. "They don't test the blood. I have to do it at home myself."

5. *Practitioners not providing the correct type and amount of information *including when new medication was prescribed.

6. *Phlebotomy skill *varied. Some nurses had no difficulty taking blood, but others had difficulty that was made worse if they did not anticipate problems, and did not like taking guidance from the patient on the best vein to use.

#### Barriers identified by patients as being related to the healthcare system

1. *The cost of drugs*. Drugs on the formulary are free. However those not on the formulary were said to be expensive.

2. *Medication supply problems *in the polyclinics. One month's supply of medication should be dispensed at a time, but because of shortages less was dispensed at times. Some patients did not see this as a problem as they eventually got all the medication despite the need for multiple visits to the pharmacy. Others were more inconvenienced. Shortages were more likely for certain medication.

3. *Poor customer service at polyclinic pharmacies*. When a prescribed drug is not available, a new prescription would be needed for an alternative drug or for the patient to obtain that specific drug from another pharmacy. "When I leave the doctor and go to the pharmacy, I like to go and ask do you have 'this' before I sit down. But some won't answer me. It don't make sense I sit down with you all for 3 hours and when I get to him say, 'I don't have it'. Then I have to go back to the doctor and get a fresh prescription and go to another pharmacy." The attitude of staff could be improved. "The civil servants of this country, they need a public relations course, they have to realise that they are doing a service. They think that we are begging them for something; I am not begging them for anything." The private sector however provided better customer service. "If you even go to the private doctor the drugs stores there...the pharmacies that you get your medicine...place like (name of a private pharmacy) you can stay home and call in your medication and send somebody for it, you don't have to go".

4. *Long waiting times in clinic to be seen*. This was a problem especially for working persons. "You have to wait very long." "I never really had the courage to go to the polyclinic because the hours I work I can't just go and sit there because I'll waste too much time there........" Others were concerned about the physical effects of waiting e.g. patients with diabetes becoming hypoglycaemic.

#### Barriers identified by patients as being related to the wider society

1. *The high cost of food*. Appropriate food (vegetables and low sugar versions of foods such as jams and ice cream) was said to be very expensive. Suggested solutions included giving discounts to patients with diabetes, people buying vegetables that were in season and therefore cheaper, and when possible growing your own food which would have the additional benefit of providing exercise.

2. *Appropriate food is not available when eating out*. This was true at a variety of settings. "Everything available at parties and public gatherings is with sugar." "Too much grease, too much salt......"

3. *Difficulty getting time off work for appointments, tests and group meetings*. Appointments, tests and group meetings often occurred on different days resulting in multiple days off work. Some persons used the sick days they were entitled to for this purpose. Others got to the clinic early so hopefully they would be able to leave at a reasonable time and get to work. However this was often not possible e.g. blood tests for diabetes often include fasting and 2-hour post breakfast glucose tests resulting in most of the morning being spent in clinic. Some felt that employers were not supportive. "They don't understand you, they don't understand your problems or they don't care." "They are not interested in your sickness and that is a big barrier that we have- taking out time."

4. *Lack of support and stigma*. "When you tell people that you are hypertensive they think that you is a different kind of person you know and some people shun you." There was concern that the general public did not always appreciate the need for dietary modification as a means of primary prevention. "People does say to me my children need sugar, people say my children need salt."

5. *Stress *in everyday life including at work was a problem. It could be dangerous. "He was also diabetic and hypertensive, and his son got into a conflict, and he got into a rage and collapsed in the yard, and he is now in St. Stephen's church yard because of this conflict." It was perceived as affecting blood glucose and blood pressure control. "If you got upset about something ....... you'd be worried if the pressure is too high and am I going to have a stroke." "I don't think doctors really believe in that, but stress plays a very high part in the level of blood sugar."

### Recommendations

Recommendations to improve care within the health care system included giving priority to patients with diabetes and hypertension at clinics, improving patient self-management skills, and showing more understanding of the difficulties patients face. For the wider society recommendations included improving access to appropriate food and exercise facilities, and for education of the general public (Table 3).

**Table 3:** Recommendations suggested by focus group participants to overcome barriers faced by those with diabetes and/or hypertension

Target	Recommendations
Self	⇒ Practice moderation, allowing occasional indulgences

Practitioners	⇒ Improved attitude ⇒ Take the whole patient into account ⇒ More empathy for patients dealing with lifestyle challenges ⇒ Provide more information

Health Care System	⇒ Better customer service especially at polyclinic pharmacies and with appointment scheduling. ⇒ Improve continuity of care ⇒ Special care and priority for patients with diabetes and hypertension comparable to that given to asthmatics ⇒ Patient education to improve self-management skills ⇒ Introduction of a patient version of diabetes and hypertension guidelines

Wider society	⇒ Lower cost "healthy" foods such as fruits and vegetables ⇒ Access to exercise facilities -affordable, available and supervised exercise programmes ⇒ Support and education groups aimed at patients and their families ⇒ Education of the general public so that they may assist people with diabetes e.g. in emergencies, and as a means of primary prevention (school programmes to influence eating habits, prominent bill boards like those for AIDS prevention)

### Guidelines

None of the patients attending the focus groups had seen any guidelines for either diabetes or hypertension despite being specifically asked about those readily available on the internet. Once guidelines were explained to them, patients were keen to have patient versions of guidelines, particularly a patient passport, giving targets for blood pressure, HBA1c etc., frequency of tests and a place to record results.

## Discussion

Audits of diabetes and hypertension primary care in Barbados have identified deficiencies [10,11]. The patients in this study have identified patient, practitioner, health care system and society related factors that may be barriers to good care and guideline adherence.

Maintaining diet and exercise programmes and taking medication consistently, were *patient factors *identified that could affect care. In the focus group study of practitioners done at the same time as this study, practitioners also noted that patients were "incapable of reversing 40 years of bad habits" [16]. Health care workers need appropriate behaviour change training so that they can enhance patients' problem solving skills and help them avoid or minimise lapses in behaviour modification.

*Practitioner factors *identified that may result in less than optimal care included the attitude of staff, problems with the continuity of care, lack of knowledge of the 'whole person' and not taking patient expectations into consideration. In contrast patients who were satisfied with care valued the rapport they had with their doctor, the fact that their expectations were met and that adequate information was shared with them. Many consultation models stress the importance of taking patient ideas, concerns and expectations, as well as the whole person into account [17,18]. A person-focused interaction style compared to biomedical and high physician control styles is associated with higher patient reported quality of primary care [19]. "Whole person" knowledge of patients and the patients' trust in their physician are associated with adherence [20]. In trying to improve the primary care of diabetes and hypertension, attention therefore needs to be given to promoting a patient-centred approach and not only to the biomedical aspects of care often stressed by guidelines and audits. Making patients feel more welcome and respected at clinics would enhance the way people feel about the health care experience.

Several *health care system factors *were identified. Many of these resulted in patients having to spend excessive time accessing care. The long waiting times to be seen after arriving at the clinic and a further waiting time to get prescribed medication from the pharmacy hamper access because of inconvenience, possible economic cost to the patient, and the difficulty experienced by some patients in getting sufficient time off work. Medication shortages at the public sector pharmacies could also act as a time related barrier. Many of these barriers were identified by the practitioner focus group study [16], showing that staff were aware of these issues. Solutions could include a better appointment system at polyclinics to cut down on waiting time and better customer focus at public sector pharmacies. The latter could include allowing patients to drop off prescriptions and collect the medication later when it was dispensed, patients being able to phone the pharmacy when repeat medication was needed and have someone collect it for them if necessary, better stocking of medication to avoid shortages, and when medication was in short supply a better system to alert patients and a more efficient system to provide them with a prescription to get the medication elsewhere. More convenient opening hours would also reduce the difficulty in getting time off work.

The cost of drugs was given as a barrier to care, but an appropriate range of medication is available in Barbados at no cost to the patient to treat most persons effectively. While some of the newer more expensive brand name drugs, to which the focus group participants may have been referring, may be beneficial for the management of some patients, providing them at no cost could divert financial resources from more cost-effective activities.

*Society related barriers *to maintaining health reside outside the direct influence of the health care system. The high cost of food and the lack of appropriate food choices when eating out were issues identified. Public health campaigns can promote healthy, cost-effective food, and counter the perception that this should include special diabetic versions. The provision of convenient and safe places to exercise can be promoted through appropriate public policy.

Focus group attendees indicated the need for more knowledge. This included patient versions of guidelines, more information from practitioners during office visits, educational talks while waiting to be seen in clinic, and support and education groups that could involve the whole family. Attendees also suggested that education of the public would be helpful in the primary prevention of diabetes.

Having a place in the patient guidelines to record results and other information was welcomed as it would help not only with self-management, but would promote continuity in a situation where the patient was not seen by their usual health care provider. It should be noted that in Barbados patients not infrequently move between different private practitioners, between the private and public sectors, and between levels of care without referral or an adequate transfer of information. This fragments care, and is a threat to personalised whole person care that a transfer of biomedical information cannot fully correct. Integration and co-ordination of care are essential elements of good primary care [13].

Many of the issues identified e.g. difficulty changing ingrained diet and exercise behaviours, financial constraints, the need for more knowledge, and difficulties accessing care are similar to those given by patients in other countries [21-23]. However, this study has drawn attention to the barriers that are likely to be important in Barbados, as well as details and nuances that are specific to Barbados such as long waiting times at public clinics and pharmacies. These need to be explored if care is to be improved.

## Limitations

The size of some focus groups was smaller than intended. This could have influenced idea generation. However, there was a lot of similarity in issues raised between sessions, with the smaller groups being no different than the larger groups, and the last focus group produced no ideas that had not been heard before.

## Conclusions

Efforts to implement guidelines into practice, while valuable, may not be sufficient to achieve a higher standard of diabetes and hypertension primary care. A patient centred approach is needed which takes the "whole person" into account, along with a better customer service approach minimising the lost time and possibly lost wages encountered by patients when accessing care. Improved self management could be helped by a patient version of the guidelines and greater educational efforts. Implementing the recommendations will require a variety of strategies. Financial implications will differ and some will be easier to implement than others. Allowing the patient to phone the public sector pharmacy when repeat medication is needed should be straightforward. Achieving a sustained change in practitioner attitude and practice could require ongoing training, ensuring adequate staff levels, monitoring, feedback and perhaps financial incentives.

## Competing interests

The authors declare that they have no competing interests.

## Authors' contributions

OPA, AOC participated in the conception and design of the study; the acquisition, and interpretation of data; and revising the manuscript critically. PA drafted the manuscript. Both authors have read and approved the final manuscript.

## Authors' information

OPA is a lecturer in Family Medicine and deputy dean, Faculty of Medical Sciences, Cave Hill Campus, University of the West Indies, Barbados. AOC is adjunct associate professor, Department of Community Health and Epidemiology, Queen's University, Kingston, Ontario, Canada.

## Pre-publication history

The pre-publication history for this paper can be accessed here:

http://www.biomedcentral.com/1471-2296/12/135/prepub
